# First-Principles Study of Discharge Products and Their Stability for Lithium-Nitrogen Batteries

**DOI:** 10.3390/ma17102429

**Published:** 2024-05-18

**Authors:** Guoxiong Qu, Xudong Zhao, Chengdong Wei, Hongyi Zhang, Yutong Yang, Hongtao Xue, Fuling Tang

**Affiliations:** State Key Laboratory of Advanced Processing and Recycling of Non-Ferrous Metals, School of Materials Science and Engineering, Lanzhou University of Technology, Lanzhou 730050, China; 17791625338@163.com (G.Q.); xdzhao1002@163.com (X.Z.); 18893459042@163.com (C.W.); zhanghongyizhi1@163.com (H.Z.); 13020772152@163.com (Y.Y.); xueht@lut.edu.cn (H.X.)

**Keywords:** surface adsorption, stability, electronic properties, lithium-nitrogen batteries, first-principles calculations

## Abstract

Li-N_2_ batteries present a relatively novel approach to N_2_ immobilization, and an advanced N_2_/Li_3_N cycling method is introduced in this study. The low operating overpotential of metal–air batteries is quite favorable to their stable cycling performance, providing a prospect for the development of a new type of battery with extreme voltage. The battery system of Li-N_2_ uses N_2_ as the positive electrode, lithium metal as the negative electrode, and a conductive medium containing soluble lithium salts as the electrolyte. In accordance with its voltage-distribution trend, a variety of lithium-nitrogen molecule intermediates are produced during the discharge process. There is a lack of theoretical description of material changes at the microscopic level during the discharge process. In this paper, the first-principles approach is used to simulate and analyze possible material changes during the discharge process of Li-N_2_ batteries. The discharge process is simulated on a 4N-graphene anode substrate model, and simulations of its electrostatic potential, Density of States (DOS), HOMO (Highest Occupied Molecular Orbital) and LUMO (Lowest Unoccupied Molecular Orbital) aspects confirm that the experimentally found Li_3_N becomes the final stabilized product of the Li-N_2_ battery. It can also be seen in the density of states that graphene with adsorption of 4N transforms from semiconducting to metallic properties. In addition, the differential charge also indicates that the Li-N_2_ material has a strong adsorption effect on the substrate, which can play the dual role of electricity storage and nitrogen fixation.

## 1. Introduction

Among various energy-storage devices, batteries are the key supporting technology for the energy, information, and transportation revolutions [1], offering the advantages of high efficiency and availability of different forms [2]. The energy density of rechargeable batteries has been increasing from nickel–cadmium and nickel–metal hydride batteries to Li-ion batteries. As a result, these energy-storage devices have been widely adopted in many smart devices [3]. In battery systems, metal–air batteries have some advantages in comparison with other types of batteries. On the one hand, they have higher safety and higher energy density [4]. On the other hand, the use of gases such as oxygen, carbon dioxide, and nitrogen from ambient air as a cathode source has the additional advantage of significantly reducing the cost and weight of the single battery [5]. Li-O_2_ batteries have been of interest for many years due to their high theoretical weight energy and high energy density [6,7]. In theory, a metal–air battery has the potential to provide a car with comparable power and speed with respect to gasoline. One of the key reasons for its high theoretical energy density is the movement of lithium ions between battery electrodes [8,9]. Unlike traditional batteries, metal–air batteries can increase the weight energy of the battery without combining heavy metals with lithium [10].

At present, in the charge/discharge process of lithium-ion batteries, there are mainly problems such as the low conductivity of discharge products limiting the utilization of active substances and the multiplication performance of the battery. In the selection of cathode materials, the α-type two-dimensional layered structure has been attracting much attention [11], which is very suitable for the embedding of lithium ions and can adapt to the high-current charging and discharging process. Two-dimensional graphene materials have the advantages of high electrical conductivity, high pore capacity, high electrochemical stability, etc. By designing a suitable pore structure or using a flexible carrier, it can effectively promote the reaction between Li^+^ and small molecules in the electrode as well as cope with volume expansion during the discharge process [12], which greatly improves the electrochemical performance of lithium-ion batteries, and is currently widely used in lithium-ion battery anode materials represented by lithium-sulfur batteries.

Zhang et al. [13] first proposed the concept of the Li-N_2_ battery and investigated it experimentally. The Li-N_2_ battery is a new battery system, which is a secondary energy-storage device with porous carbon cloth as the positive electrode, lithium metal foil as the negative electrode, and a conductive medium containing soluble lithium salts as the electrolyte. Experimental results [13] provided that the open circuit voltage and energy density of the Li-N_2_ battery were 0.54 V and 1248 Wh∙kg^−1^. Li-N_2_ battery and Li-air battery belong to the same metal–gas battery systems, and there should be a lot of similarities between the two in terms of battery structure and energy conversion.

The N_2_ reduction reaction could be used to synthesize the high-value chemical NH_3_. It was important for renewable electricity generation because of its mild operating conditions and abundance of reagents [14]. In addition to the synthesis of NH_3_ by the N_2_ reduction reaction, M-N_2_ (M: metal) batteries, which have been researched and developed in recent years, present a distinctive idea by combining N_2_ conversion and energy storage at the same time [15,16]. The M-N_2_ batteries reported so far were mainly divided into two categories: aqueous batteries represented by Zn-N_2_ and organic-based M-N_2_ batteries represented by Li-N_2_ battery [16,17]. Among them, the Li-N_2_ battery system is only an energy-conversion device, distinguishing it from other secondary batteries in that the active substance nitrogen required for the electrode reaction comes from the outside air. Most importantly, Li-N_2_ batteries not only provided the basis and technological advancement as electrochemical energy-storage devices, but also provided a reliable N_2_/Li_3_N cycling process for invertible N_2_ immobilization, which was expected to meet the future target requirements of high performance and sustained durability [18].

Zhang et al. [13] found that although N_2_ could be fixed in Li-N_2_ batteries under certain environmental conditions, the instability and hygroscopicity of the discharge product Li_3_N would cause the charging efficiency of the batteries to drop and become irreversible. In addition, the low efficiency and irreversibility of Li-N_2_ batteries was also influenced by the strong nonpolar N≡N covalent triple bond with high ionization energy. To solve the above problems, the researchers proposed using graphene as an anode for Li-N_2_ batteries. They used the porous and folded structure of thin graphene nanosheets [19], which facilitated faster migration of lithium ions and helped inhibit parasitic reactions during the deposition/dissolution process. In addition, in situ generation of Li_3_N and LiOH in a pure N_2_ atmosphere further improved the depletion and volume change of the Li metal anode. This approach was found to significantly enhance the cyclic stability and rechargeability of Li-N_2_ batteries.

According to Ma et al. [20], a certain ratio of vaporized N_2_ and H_2_O was used as the reactant, which entered the inner surface of the cathode through a gas diffusion layer. Ru nanoparticles were selected as suitable and adaptable electrocatalysts for nitrogen reduction based on the fundamental idea of continuous lithium-mediated nitrogen reduction in the Li-N_2_ battery system. N_2_ and H_2_O were then continuously energized into the cathode gas diffusion electrode and maintained for a specific amount of time to reduce reactants and produce ammonia.

Research on Li-N_2_ batteries is still in its early stages. While some researchers have conducted some studies on Li-N_2_ batteries, there is a lack of comprehensive research on the relationship between battery materials and performance. In particular, there is a scarcity of studies that use computational simulation methods and first-principles methods to speculate on material changes and reaction mechanisms during loading and discharging. In this paper, the discharge products that may be generated during the discharge process of Li-N_2_ batteries are computationally analyzed to judge the stability of adsorption, the possibility of power storage, and nitrogen fixation.

## 2. Computational Details

All the computational work in this paper was based on the first-principles computational approach of density-functional theory (DFT) and processed in the VASP (6.3.2) software package [21,22]. The generalized Perdew–Burke–Ernzerhof (PBE) functional modification of the exchange-correlation energies [23] was used, the generalized gradient approximation (GGA) was chosen for the generalization method [24], and the interactions between ions and electrons were described via the projective affixed plane wave (PAW) method [25]. The DFT+D3 study method was used in the input file INCAR for more accurate results in order to eliminate errors arising from overestimation of the repulsive forces between electrons due to the inability of the DFT to reasonably counteract the self-interactions of the electrons [26]. For geometrical relaxation, an energy cut-off of 460 eV and the 4 × 4 × 1 Monkhorst-Pack K-point mesh were selected, the electron self-consistency convergence criterion was less than 5.0 × 10^−2^ eV, and the ion chirality convergence criterion was less than 1.0 × 10^−6^ eV. The BFGS (Broyden-Fletcher-Goldfarb-Shanno) was used to calculate the electron wave function and charge density using an energy-minimization method to find the ground state and optimize all crystal structures [27]. To avoid the effect of periodic boundaries, we added a vacuum layer of 20 Å thickness to the adsorption substrate. The visualization software VESTA (ver.3.5.7) was used for property analysis during the study [28].

## 3. Results and Discussion

### 3.1. Substrate Modeling

**Figure 1:**
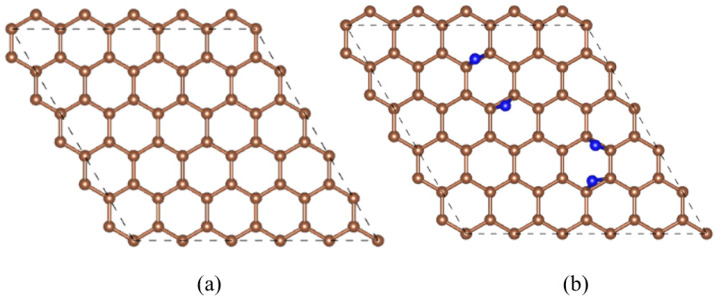
(**a**) Graphene planar surface; (**b**) optimized 4N-graphene structural model (brown for C atoms, blue for N atoms).

In this paper, a graphene anode (graphene structure shown in Figure 1a) is used as a conductive material in Li-N_2_ batteries and as a site for chemical reactions between Li and N_2_ molecules. Figure 1b shows a 4N-graphene model of 4 N atoms adsorbed on 50 C atoms on which subsequent Li adsorption will be performed. This model is a convenient starting point for the adsorption calculation of discharge products on it. The graphene carbon cloth chosen is characterized by its periodicity. Considering the periodic boundary effect of graphene, we chose a model that can represent the whole macroscopic model. Secondly, we considered the size effect and chose the most suitable modeling system available, as it provides good accuracy for the system without requiring high computational resources.

### 3.2. Structure and Properties of Li Polynitrogen Free Molecules

In the normal charging and discharging process of Li-N_2_ batteries, it was known that the reactants are lithium metal and nitrogen, and the product is Li_3_N deposited on the carbon surface [13]. Although the total reaction formula is 6Li + N_2_ = 2Li_3_N, the generation of Li_3_N is not a one-shot process in the actual situation; there should be some intermediate reactions and products. Li-N_2_ battery research is in its early stages, and various studies have not gone far enough to characterize the products of the various reaction stages. Before studying the adsorption of lithium atoms, various possible free molecules formed by lithium and nitrogen need to be investigated as a basis for lithium adsorption research.

Ma et al. [29] predicted the kinetic mechanism of the Li-N_2_ battery using Ru as catalyst and gave the corresponding reaction paths. We would like to speculate on and simulate the possible intermediates in the reaction path of Li-N_2_ batteries, and calculate their structures and physicochemical properties. For example, as a result of nitrogen with high mobility, it would usually appear in the initial discharge process in the intermediate surface. Lithium ion and nitrogen atoms may form a small molecule structure, similar to various small molecule products in ammonia synthesis under electrochemical action. The structures and properties of these small molecules have important implications for exploring the reaction mechanism of Li-N_2_ batteries. Lithium and hydrogen are elements of the same main group, so intermediate products of chemical synthesis of ammonia have important implications for lithium-nitrogen compounds.

Potential reaction pathways and Li-N_2_ battery intermediates including LiN_2_, Li_2_N_2_, Li_3_N_2_, Li_4_N_2_, Li_2_N and Li_3_N adsorbed on Ru surface were also given in the work of Ma et al. [29]. We calculated the possible chemical reactions of several free nitrogen and lithium molecules adsorbed on graphene, among which are LiN, Li_2_N, Li_3_N, Li_3_N_2_. By analyzing the configuration, density of states, and molecular orbitals of these small molecules, it is convenient for future research on the mechanism of operation of Li-N_2_ batteries with graphene as the cathode material.

**Figure 2:**
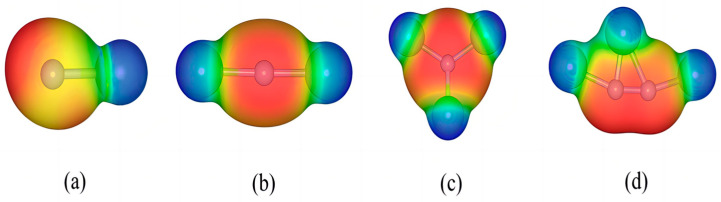
Electrostatic potential of (**a**) LiN, (**b**) Li_2_N, (**c**) Li_3_N, and (**d**) Li_3_N_2_ molecule.

The four small molecules LiN, Li_2_N, Li_3_N, and Li_3_N_2_ were modeled, and their structures were optimized via VASP (6.3.2) software. Generally, bond strength can be expressed as the bond length between two atoms, and the shorter the length of the same bond type, the stronger the degree of bond between the two atoms. The optimized results of the constructed model show that the bond length of LiN is 1.439 Å, which indicates that the LiN molecule has a strong Li-N bond. In addition, the Li-N bond lengths of three other molecules are generally in the range of 1.7–1.8 Å, including the bond length of Li_3_N, the most stable discharge product of the Li-N_2_ battery. As a result, it is difficult to convert small lithium-nitrogen molecules into lithium-nitrogen compounds without catalytic assistance or energy stimulation (for example, high temperature). In accordance with Figure 2, we can learn some information about the electron density distribution of molecules, in which the red area (corresponding to around the N atom) and the blue area (corresponding to around the Li atom) indicate the electron-accumulation area and the electron-deficiency area. Accordingly, positively charged particles interact strongly with the electron-accumulating region due to its nucleophilic activity, whereas negatively charged particles interact strongly with the electron-deficient region due to its electrophilic activity. By comparing the surface electrostatic potential maps of the four intermediates, it can be found that the area around the N atom in all structures is red; the electrostatic potential is negative; then it has a strong interaction with positively charged particles and is easily approached by them. In contrast, the area around Li atoms is blue and easily approachable with negatively charged particles.

**Figure 3:**
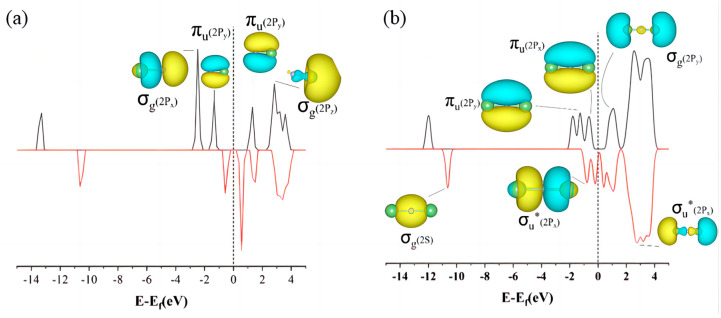
HOMO, LUMO electron distributions and density of states of (**a**) LiN and (**b**) Li_2_N molecules.

**Figure 4:**
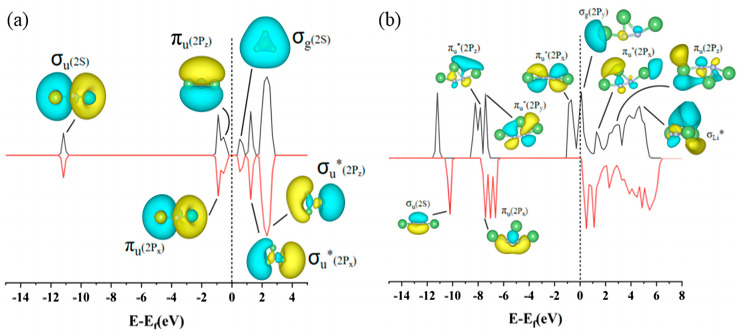
HOMO, LUMO electron distribution and density of states of (**a**) Li_3_N and (**b**) Li_3_N_2_ molecules.

The density of states (DOS) map not only reflects the distribution of electrons in individual orbitals, but also reveals important information such as chemical bonding, conductivity, and so on. According to the energy band theory, it can be seen that the lowest unoccupied molecular orbital (LUMO) exhibits a conduction band, which is on the right side of the Fermi energy level. The highest occupied molecular orbital (HOMO) exhibits a valence band, which is on the left side of the Fermi energy level. On comprehensive analysis of the density of states Figure 3 and Figure 4, it is found that due to the coincidence of the electron orbits of Li and N atoms, Li_3_N molecules have the same distribution of spin electrons and spin electron energy levels. The DOS diagrams are symmetrical up and down. This also explains why Li_3_N is the final product of the Li-N_2_ battery. From the inconsistency in the energy level distributions of spin-up electrons and spin-down electrons, we can infer that the electronic orbitals of small molecules such as LiN and Li_2_N are not occupied to full capacity (despite the relatively high bonding strength of LiN, the bonding type is not a Li-N triple bond, and empty orbitals on LiN molecules would force LiN molecules to shift to other LiN compounds). In addition, the highest occupied molecular orbital (HOMO) of these two small molecules is in the bonding state (π_u_), and when the electrons of these two small molecules are transferred from this orbital to the electrophilic substrate, these two small molecules will be in an unstable state and they are easier to be converted into other small molecules. Li_3_N_2_ has two Li-N bonds of different lengths due to poor orbital matching between the intermediate lithium atom and the two nitrogen atoms. The highest occupied molecular orbital (HOMO) of Li_3_N_2_ is the antibonding state (π_u_*); the bonding strength of the two nitrogen atoms is enhanced when electrons are transferred from this orbital to the electrophilic substrate, suggesting that Li_3_N_2_ is more inclined to be converted to the series of small molecules of Li_x_N_2_. In summary, the poor matching of the electron orbitals of lithium and nitrogen atoms of small molecules such as LiN, Li_2_N, and Li_3_N_2_ results in poor structural stability of these small molecules, which are all eventually converted into the relatively more stable Li_3_N.

### 3.3. Calculation of Electrode Potentials of Li-N Substances on Graphene

**Figure 5:**
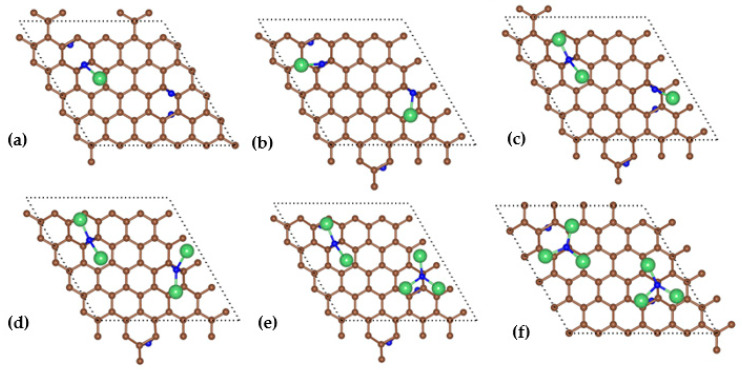
Structures of (**a**) LiN_4_, (**b**) LiN_2_, (**c**) Li_3_N_4_, (**d**) LiN, (**e**) Li_5_N_4_, (**f**) Li_3_N_2_ after adsorption on graphene surface. (brown for C atoms, green for Li atoms, blue for N atoms).

The voltage of a battery is determined by the difference between the electronic potentials of the positive electrode and that of the negative electrode. The potential of positive and negative electrode materials is usually generated by the reaction of reactants and products with lithium electrodes. The standard lithium electrode is often used as the reference electrode. We used the first principles to simulate the reaction process between lithium atoms and nitrogen atoms embedded in the discharge process of the Li-N_2_ battery with graphene common material as the positive electrode, and to investigate the generation of the final discharge product Li_3_N and the voltage change during the discharge process. We first explore the microscopic products and reaction mechanisms in the discharge process of Li-N_2_ batteries (as shown in Figure 5), then explain the changes in the calculated and experimentally observed discharge products and voltage values in turn.

**Figure 6:**
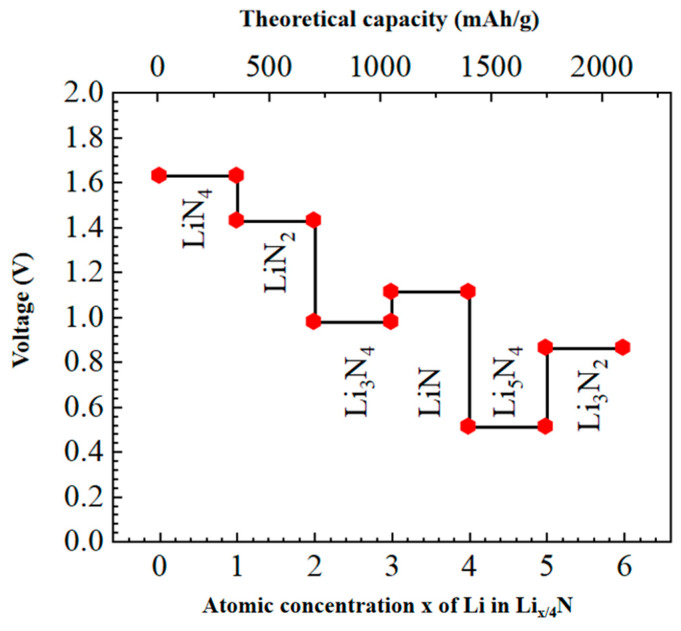
Voltage variation during discharge of Li_x_N_4_ (1 ≤ x ≤ 4).

It is found that with the increase of the number of Li atoms embedded in the anodic graphene, its electrode potential as a whole gradually decreases, and the discharge capacity of the battery gradually increases. The maximum discharge capacity and initial voltage of the battery are 2311 mAh/g and 1.61 V, respectively. The final discharge voltage of the battery is 0.82 V. The numerical simulation is basically in line with Zhang’s theoretical or numerical results [13]. In general, the discharge capacity increases as its discharge voltage decreases. However, voltage anomalies often occur during battery discharge, which is mainly due to the fact that different discharge products are often formed during the discharge process, resulting in abnormal voltage changes. The voltage of the discharge process with the number of lithium ions is shown in Figure 6: anode embedded in 1 Li atom, the discharge product is LiN, at this time the voltage is maximum; embedded in 2–3 Li atoms, the discharge product is LiN + LiN and LiN + Li_2_N, the voltage is reduced; the anode embedded in 4 lithium atoms, the discharge product is Li_2_N + Li_2_N, the voltage rises by 0.13 V; when the positive electrode is embedded with 5 lithium atoms, the discharge product is Li_2_N + Li_3_N, and the voltage decreases by 0.59 V; when 6 lithium atoms are embedded, the discharge product is Li_3_N + Li_3_N, and the voltage of the positive electrode increases to 0.86 V. Analyzing the above process, it is found that it is mainly due to the formation of Li_2_N and Li_3_N discharge products during the discharge process. From a thermodynamic point of view, the formation of Li_3_N is more conducive to the stability of the system. However, when the discharge product is stable Li_3_N, the discharge voltage and capacity increase. Therefore, we believe that there is a possibility of overcharging the battery during Li_3_N formation. Compared to Figure 1, the atomistic positions in Figure 5 have changed, but the basic structures of the crystal lattices remain unchanged according to the periodic boundary condition.

The calculation results in Figure 6 show that the voltage generally decreases with the increase in discharge capacity, but the local position of the curve appears to be a stepped voltage. In addition to the material reasons mentioned above and the disregard for volume work and entropy change, the chemical reaction generating various small molecules in the experiment can occur at any time. In this way, the actual discharge process is the average effect of the generation of individual small molecules, which makes the discharge curve become continuous and smooth. The first-principle simulations cannot account for the likelihood of different types of reactions occurring at a specific moment. However, the change in substance ultimately indicates the trend in electrode potential. This trend may include minor fluctuations in the electrode potential of the generated substance, but the overall tendency for the final electrode potential to reach a minimum remains unchanged. In addition, as the discharge reaction progresses and the number of cycles increases, the cathode material undergoes changes in both structure and properties. Continued product accumulation also leads to changes in the adsorption of atoms and the fresh surface of the reaction, ultimately affecting the voltage capacity curve.

### 3.4. Linear Correlation between Adsorption Energy and Charge

**Figure 7:**
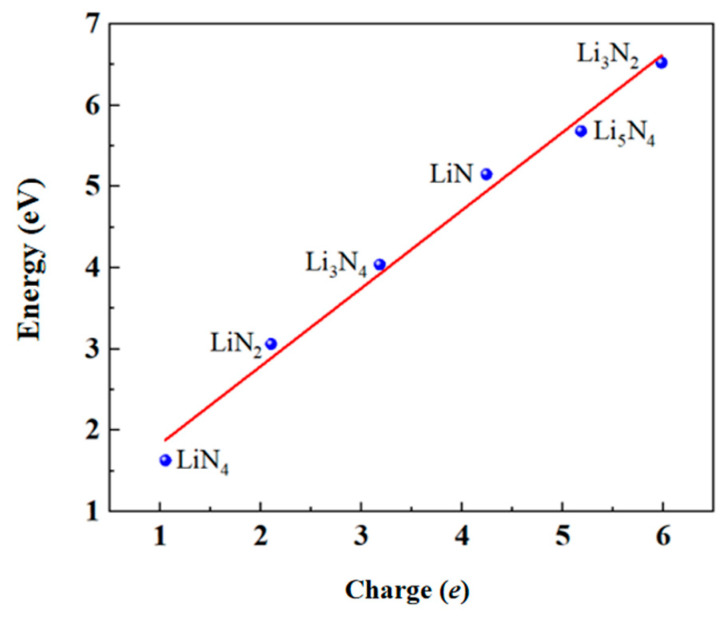
Fitting of the relationship between the adsorption energy and the charge transfer of Li atoms in the system.

The adsorption energies of the six Li atom adsorption systems are calculated and shown in Figure 5, 1.63, 3.06, 4.04, 5.15, 5.66, and 6.52 eV, taking the adsorption energies as their absolute values. The larger the adsorption energy, the more stable the adsorption system. The more Li atoms adsorbed on the graphene, the larger the adsorption energy. The corresponding total Mulliken charges Q of the adsorbed Li atoms were calculated to be +1.05, +2.10, +3.18, +4.24, +5.18, and +5.98 |e|, which are presented in Figure 7. It is widely known that the charge of each adsorbed Li in the structure undergoes a charge transfer to the substrate; the adsorption energy of the system can be fitted to a straight line with the amount of charge transfer; its relational equation is:

*E* = 0.87 + 0.96*Q* (1)

where the fitting error of *E* is basically within ±0.2 eV, indicating that the dispersion of individual data from the overall data is small (correlation coefficient of 0.989), and the data correlate well linearly. Furthermore, it suggests that the larger the absolute value of the adsorption energy, the more pronounced is the charge transfer of the system. There is a direct relationship between charge transfer and surface adsorption; it may serve as a new descriptor to characterize the important phenomenon of adsorption.

In accordance with the linear fitting Equation (1), our results demonstrated that the adsorption energy and the coulombic energy are closely involved with each other in a power-of-one linear relationship, but the coulombic energy should be more proportional to the power-of-two of the transferred charge. We conjecture that the power anomaly of the fitted equation may come from interactions such as attitude adjustment of the molecules with the substrate, molecular spacing, van der Waals, etc., which requires further analysis.

### 3.5. Electrical Properties: Density of States and Differential Charge

The electronic structure of the 4N-graphene system will change after Li adsorption, and its electronic properties will change accordingly. In order to further investigate the changes in electronic properties before and after adsorption, the density of 4N atoms before adsorption, graphene substrate, nitrogen/graphene composites, and the adsorption system with six types of atomic distributions (LiN_4_, LiN_2_, Li_3_N_4_, LiN, Li_5_N_4_, and Li_3_N_2_) formed by sequentially adsorbing Li atoms were further calculated.

**Figure 8:**
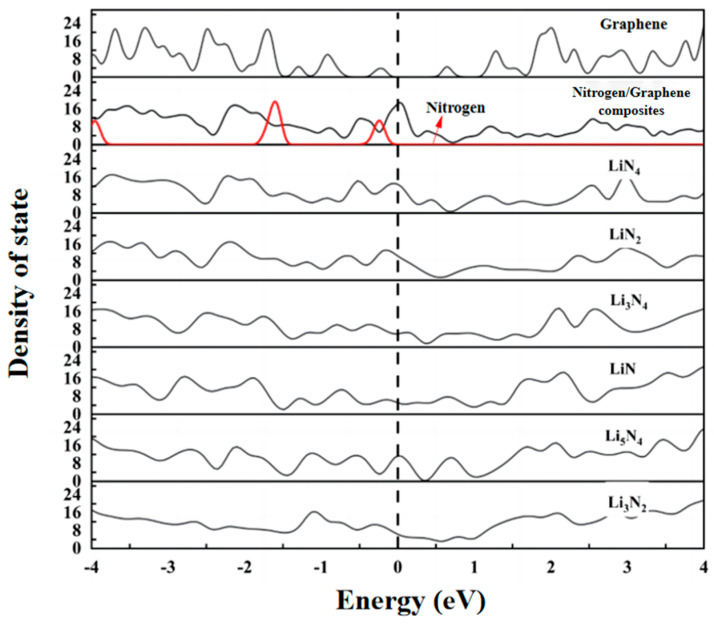
Density of states of N_2_ molecules, graphene, nitrogen/graphene composites, and Li polynitrogen molecules.

We can see the results in Figure 8. Graphene used in the calculations is semiconducting in nature with a band gap of 0.5 eV. Graphene and N atoms alone did not show any electronic states at the Fermi energy level. However, when N atoms were adsorbed on the graphene surface, the nitrogen/graphene composite material showed significant electronic states at the Fermi energy level, indicating that the matrix material has better electrical conductivity as a cathode material for batteries. We then sequentially adsorbed Li atoms before the formation of six Li-N distributions on the surface. The Fermi energy level value of the total density of states of the system after this adsorption is reduced, the most with the formation of LiN, and the least with LiN_4_. All adsorption improves the conductivity of graphene, which is beneficial for use as a positive electrode.

Differential charge density can visualize the charge transfer of an adsorption or chemical process, and is mostly used to study charge redistribution due to interactions between molecules, clusters, solid materials, and molecules and solid materials. The differential charge is calculated as follows:

(2) $$\Delta {\rho =\rho }_{{\mathrm{nLi}/(N}_{2}/\mathrm{graphene})}{\;-\;\rho }_{N_{2}/\mathrm{graphene}}{\;-\;\rho }_{\mathrm{nLi}}$$

*ρ_nLi/(N_2___/graphene)_* represents the total charge density of graphene after nitrogen fixation and chemical inversion with Li atoms, *ρ_N_2___/graphene_* represents the charge density of graphene after nitrogen fixation, *ρ_Li_* represents the total charge density of adsorption of different numbers of Li atoms, and *n* is the number of Li atoms in the system. Differential charge is the determination of electron gain and loss from atom to atom, and the resulting orbital hybridization and chemical bonding as judged by the area of overlap between electron-gaining and electron-losing regions.

**Figure 9:**
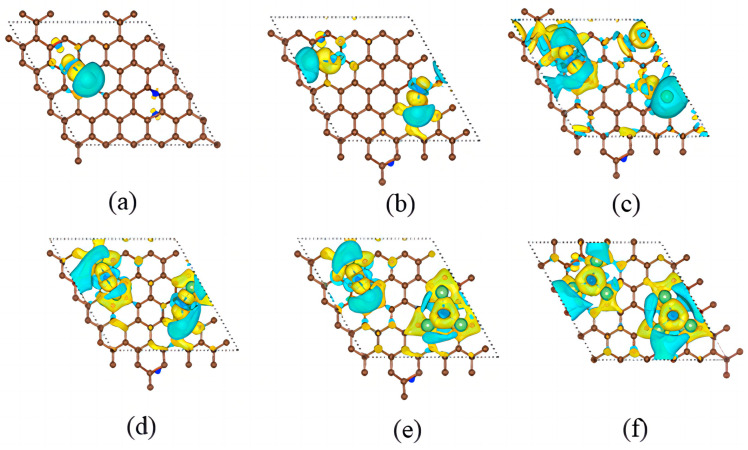
Differential charges of (**a**) LiN_4_, (**b**) LiN_2_, (**c**) Li_3_N_4_, (**d**) LiN, (**e**) Li_5_N_4_, (**f**) Li_3_N_2_ adsorbed on graphene surface.

The top and side views of the differential charge for the adsorption models (Figure 5) are shown in Figure 9, where the yellow area reveals electron accumulation and the blue area reveals electron dissipation. When the yellow or blue regions are large, it reveals that there is more charge transfer at that location, then the interaction between the two atoms at that location is stronger. In previous calculations, it was found that the adsorption energy gradually increases with the increase in the adsorbed number of Li atoms, and the charge transfer also increases. At the same time, the electron transfer area of the differential charge region increases. From Figure 9, it can be observed that there is a strong interaction between Li atoms and surfacial N atoms, indicating that Li atoms are adsorbed on the surface of the positive electrode during the discharge process of the Li-N_2_ battery, which in turn promotes the nitrogen fixation effect of the battery.

## 4. Conclusions

In this paper, we established a 4N-graphene atomic model as the cathode substrate model for first-principle calculations and simulated and then analyzed the possible material changes that may occur during the discharge process of Li-N_2_ batteries. Firstly, by calculating the surface electrostatic potentials, the LUMO and HOMO electron distributions of the free molecules of Li polysulfide, we analyzed the stability of the free molecules and found that Li_3_N is the most stable and reasonable of several possible molecular structure products, and the results were in line with the reference conclusions [20] for the final products in lithium-nitride batteries. Secondly, the adsorption energy is linearly related to the Mulliken charges of Li atoms on the substrate, and the larger the adsorption energy of the system, the more stable the adsorption system is. In addition, a cell voltage–capacity curve was derived; the simulation results show that the number of lithium atoms in graphene increases and the battery discharge capacity increases. Meanwhile, the differential charge also shows that the Li-N system has strong mutual adsorption with the graphene substrate, and its dual role in electricity storage and nitrogen fixation is possible due to the strong charge transfer, suggesting that rechargeable Li-N_2_ batteries offer a promising green candidate for N_2_ fixation and have the ability to provide an advanced N_2_/Li_3_N cycling method for next-generation energy-storage systems [20]. Most importantly, six intermediates, LiN_4_, LiN_2_, Li_3_N_4_, LiN, Li_5_N_4_, and Li_3_N_2_, appear during the discharge process of the Li-N battery, and these small molecules have poor structural stability due to the poor match between the Li and N atomic electron orbitals, and they are eventually converted into the relatively stable Li_3_N [13], which is the final product. The Li-N_2_ battery system as an energy-conversion device realizes reversible N_2_ fixation, which is expected to meet future goals of high performance and sustained durability.

## Data Availability

Data are contained within the article.
